# The Role of Metabolic Plasticity of Tumor-Associated Macrophages in Shaping the Tumor Microenvironment Immunity

**DOI:** 10.3390/cancers14143331

**Published:** 2022-07-08

**Authors:** Md Nabiul Hasan, Okan Capuk, Shivani M. Patel, Dandan Sun

**Affiliations:** 1 Department of Neurology and Pittsburgh Institute for Neurodegenerative Disease, University of Pittsburgh, Pittsburgh, PA 15260, USA; capuk@pitt.edu (O.C.); smp192@pitt.edu (S.M.P.); 2 Veterans Affairs Pittsburgh Health Care System, Geriatric Research Educational and Clinical Center, Pittsburgh, PA 15240, USA

**Keywords:** immune cell, myeloid cells, macrophage, TAM, metabolism, immune response, immunotherapy, proton pumps

## Abstract

**Simple Summary:**

Tumor associated macrophages (TAMs) support disease progression by providing tumor cells cytokines and chemokines necessary for malignant growth and nutrient support. In the tumor microenvironment (TME), TAMs often rewire their metabolism to reprogram their functions, which includes changes in the production of angiogenic factors and cytokines. These promote the pro-tumor function of the TAMs as well as the blunt T cell’s effector function, which at least, in part, explains the failure or sub-optimal efficacies of TAM-directed therapeutic approaches and immunotherapies. In recent times, much of these are attributed to the altered metabolism of the TAMs in the TME. Therefore, understanding the metabolic changes of the myeloid cells of the TME is an essential step in developing novel therapeutic approaches targeting immune cell metabolism. This review article aims to summarize the recent findings on the metabolism of TAMs, and how the altered metabolism of these innate immune cells shapes the tumor microenvironment and the anti-tumor immunity.

**Abstract:**

Cancer cells possess a high metabolic demand for their rapid proliferation, survival, and progression and thus create an acidic and hypoxic tumor microenvironment (TME) deprived of nutrients. Moreover, acidity within the TME is the central regulator of tumor immunity that influences the metabolism of the immune cells and orchestrates the local and systemic immunity, thus, the TME has a major impact on tumor progression and resistance to anti-cancer therapy. Specifically, myeloid cells, which include myeloid-derived suppressor cells (MDSC), dendritic cells, and tumor-associated macrophages (TAMs), often reprogram their energy metabolism, resulting in stimulating the angiogenesis and immunosuppression of tumors. This review summarizes the recent findings of glucose, amino acids, and fatty acid metabolism changes of the tumor-associated macrophages (TAMs), and how the altered metabolism shapes the TME and anti-tumor immunity. Multiple proton pumps/transporters are involved in maintaining the alkaline intracellular pH which is necessary for the glycolytic metabolism of the myeloid cells and acidic TME. We highlighted the roles of these proteins in modulating the cellular metabolism of TAMs and their potential as therapeutic targets for improving immune checkpoint therapy.

## 1. Introduction 

In addition to neoplastic cells, the tumor microenvironment (TME) is composed of non-neoplastic cells, such as immune cells, endothelial cells, fibroblasts, etc. Even though immune cells are the most abundant non-neoplastic cell types in the majority of solid tumors, the TME is often immunosuppressive [1,2]. The innate immune cells in tumor tissues mainly include tumor-associated macrophages (TAMs) and myeloid-derived suppressor cells (MDSCs), which account for more than half of the non-tumor cells of the TME and are generally associated with a worse cancer prognosis [3,4]. Tumor cells have developed mechanisms to orchestrate these myeloid cells’ phenotypes to promote tumor growth by regulating angiogenesis, stimulating metastasis, and suppressing immune function [5,6]. In addition, myeloid cells are regulated by microenvironmental factors such as chemokines, cytokines, growth factors, as well as metabolites [7,8]. However, several myeloid cell-directed therapeutic approaches by targeting CSF-1R inhibition have been tried with little or no success, especially for glioma, an immunologically cold tumors [9,10]. Therefore, alternative approaches for the target of myeloid cells are necessary. 

In response to diverse stimuli, immune cells undergo metabolic reprogramming to support their differentiation, proliferation, and pro-inflammatory effector functions [11]. However, these metabolic alterations are context-specific and cell-type-dependent [12]. Cancer cells use aerobic glycolysis to meet their energy demands even in the presence of oxygen, a phenomenon termed “Warburg metabolism”. Cancer cells predominantly utilize glucose for glycolytic metabolism and secret lactate [13]. Cancer cells can harness these metabolic byproducts to hijack the function of immune cells to promote tumor progression. This is true for the glycolytic cancer cell-secreted lactate which turns the immune cells into an immunosuppressive phenotype [14]. Additionally, all the immune cells of the TME compete for the limited nutrients present [15]. All these interconnected factors shape the metabolism of the evolving neoplasm [16]. A better understanding of the mechanism of immune cell metabolism and its influence on cancer immunotherapies is of paramount importance since the recent advances in the immunotherapies such as immune checkpoint blockade therapy in treating pancreatic cancer, sarcoma, and melanoma [17,18,19]. Similar to T cells, myeloid cells undergo robust metabolic changes upon stimulation and activation. Myeloid cells are highly heterogeneous and can exhibit heterogeneous metabolic features, depending on the stimuli and the TME they reside in. These diverse metabolic responses can give rise to many polarization states and phenotypes, thus controlling the downstream immune responses [11]. TAM subsets not only differ in their phenotypes regarding cytokine and surface marker expression, but also in their metabolism [20]. Here, we reviewed the main metabolic pathways used by myeloid cells of the TME in shaping the evolution of the neoplasm. We also discussed some of the current strategies to improve the efficacy of cancer immunotherapies or enhance T cell cytotoxic function by regulating myeloid cell metabolism.

## 2. Function of Tumor-Associated Macrophages (TAM) in TME 

Myeloid cells are a heterogeneous [3,21] population of innate immune cells that constitute more than 70% of all immune cell populations in the TME [22]. Common myeloid progenitor cells give rise to macrophages, myeloid-derived suppressor cells (MDSC), granulocytes, dendritic cells (DC), and neutrophils [23,24]. These cells are associated with varying degrees of tumor-promoting and anti-tumor functions [23,25,26,27]. The details of the myeloid cell types and their roles have been described in a review by Haas et al. [26]. In the current article, we focused on the discussion of TAMs that include infiltrated peripheral myeloid cells (macrophages) and brain-tissue-resident macrophages known as microglia. 

The cytokines and the molecules responsible for the recruitment and adhesion of the macrophages produced by the tumor microenvironment recruit macrophages to the tumor microenvironment [28,29]. It has been proposed that the recruitment and differentiation of the macrophages are related to local anoxia, inflammation, and the presence of high levels of lactic acid [14]. The CC chemokines, such as CCL2, CCL11, CCL16, and CCL21, are major determinants of macrophage infiltration and angiogenesis in the cancer of the breast, lung, esophagus, ovary, and cervix [28]. Most brain-tissue-resident macrophages (microglia) arise from the yolk sac of the embryo, apart from those in the intestine [30,31]. In glioma, the microglia originate from the embryonic yolk sac precursor [32]. In contrast, macrophages involved in the response against pathogens are derived from the bone marrow (BM) [5,26]. 

### 2.1. TAM in Promoting Tumor Progression

The main function of the macrophages is to recognize and phagocytize antigens and present them to the T cells; therefore, macrophages are considered the bridge between innate and acquired immunity [33]. A high level of TAMs correlates with the progression of the disease in multiple cancers [34,35,36] and plays a key role in tumor initiation and progression, including tumor invasion, migration, angiogenesis, immunosuppression, and metabolic alterations [37]. TAMs produce growth factors, cytokines, and chemokines, and trigger immune exhaustion of the T cells, developing the immunosuppressive tumor microenvironment [38,39]. TAMs possess a dual function in the TME where they promote the vasculature, intravasation, extravasation, and survival of tumor cells [40]. It is well documented that TAMs interact with the cancer cells to facilitate tumor progression [41]. SPP1/CD44 signaling of the glioma-infiltrated macrophages was shown to interact with the glioma cells and induce mesenchymal glioma formation [22]. However, more importantly, TAMs even contribute to the stemness of the tumor cells in breast cancer through the TAM–tumor cell interaction [42,43]. Cx3Cr1-deficient microglia/macrophages upregulate IL1β and promote enhanced glioma growth with an enhanced stem cell phenotype [44]. By direct or indirect interactions, TAMs can exert effects on cancer stem cells (CSCs) including chemoresistance, perseverance, and the ability to differentiate [45,46]. 

### 2.2. TAMs Interaction with T Cells

**Figure 1:**
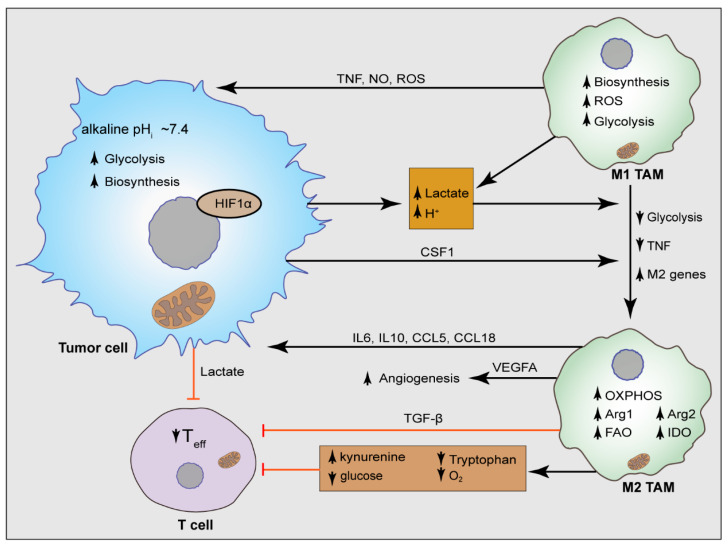
Metabolic crosstalk between TAMs and TME. Highly proliferative cancer cells use aerobic glycolysis and consume an elevated amount of glucose for bioenergetic and biosynthetic purposes, releasing lactate and H^+^ in the TME and able to release elevated CSF1. Lactate, H^+^, and CSF1 favor the polarization of M1 TAMs to immunosuppressive M2 macrophages which are characterized by the release of metabolic modulators, and immunosuppressive molecules that foster disease progression, including vascular endothelial growth factor A (VEGFA), tumor necrosis factor (TNF), C-C motif chemokine ligand 5 (CCL5), CCL18, interleukin 6 (IL6), and IL10. Moreover, lactate and glucose deprivation exerts immunosuppressive effects on effector T (T_eff_) cells. M2 TAMs synthesize abundant levels of arginase 1 (ARG1), ARG2, and indoleamine 2,3-dioxygenase 1 (IDO1), changing the metabolic landscape of the TME and secretes TGF-β which reduces effector T cell function. TNF, tumor necrosis factor; NO, nitric oxide; ARG1, arginase 1; ROS, reactive oxygen species.

Growing evidence shows that TAMs not only interact with the tumor cells, but also with the other immune cells in the TME such as T cells, and natural killer (NK) cells [47,48]. To date, the majority of the immunotherapeutic approaches are developed targeting the T cells [49]. TAM-secreted TGF-beta and IL-10 hinder the maturation of the dendritic cells (DCs), promote the expansion of regulatory T cells (Tregs), and suppress the activity of T cells and NK cells [50,51,52,53]. Additionally, TAMs express PD-L1, PD-L2, and PD-1, as well as the CTL-associated protein 4 (CTLA-4) ligands CD80 and CD86, which bind to their receptors in the CD8^+^ T cells and lead to the inhibition of the T cells’ cytotoxic function [54]. Additionally, TAM-secreted TGF-β inhibits the expression of perforin, granzyme A and B, IFNγ, and the FAS ligand [55] or CXCR3 on T cells [39], which are vital for the T cell-mediated cytotoxicity. TAMs expressing arginase, an arginine metabolizing enzyme, also affect the T cell function by creating an arginine-deprived TME and altering the T cell metabolism [54] (Figure 1). Therefore, TAM possesses a multifaceted avenue to control the T cells’ function in the TME, which is the center of most immunotherapeutic approaches. 

Activated macrophages are often classified as classical-activated M1 macrophages and alternative-activated M2 macrophages [40]. In general, M1 macrophages are the major components of host defense against infection in the context of Th1 immunity, whereas M2 macrophages support Th2 immune responses such as tissue repair and remodeling [16,56]. These terms were originated to describe the differential gene expression of the macrophages in response to interferon-γ (IFN-γ) and/or lipopolysaccharide (LPS) compared to that of IL-4 activation [57,58]. Along this line, TAMs can be classified into M1 (pro-inflammatory) and M2 (anti-inflammatory), respectively [16]. However, TAMs’ functional phenotypes in the neoplastic tissues differ between tumors or even between regions within the same tumor, and many instances do not fit the simplified M1/M2 classification [3,59]. Therefore, the M1/M2 classification is overly simplified, lacking immunological contextualization with complex/mixed phenotypes [60]. Transcriptomic analysis of the TCGA and METABRIC breast cancer datasets indicated that the M1 macrophage signature is associated with poor prognosis along with high immune activity and expression of immune checkpoint molecules [61]. These new findings indicate the ineffectiveness of simplified M1/M2 macrophage polarization characterization and warrant for a deeper understanding of different macrophage activation stages, phenotypes, and associated cellular functions in the TME. Therefore, in the next section of this review, we summarized the relationships between TAM metabolism and their tumor-promoting and/or anti-tumor functions. 

## 3. Energy Metabolism of TAMs 

**Figure 2:**
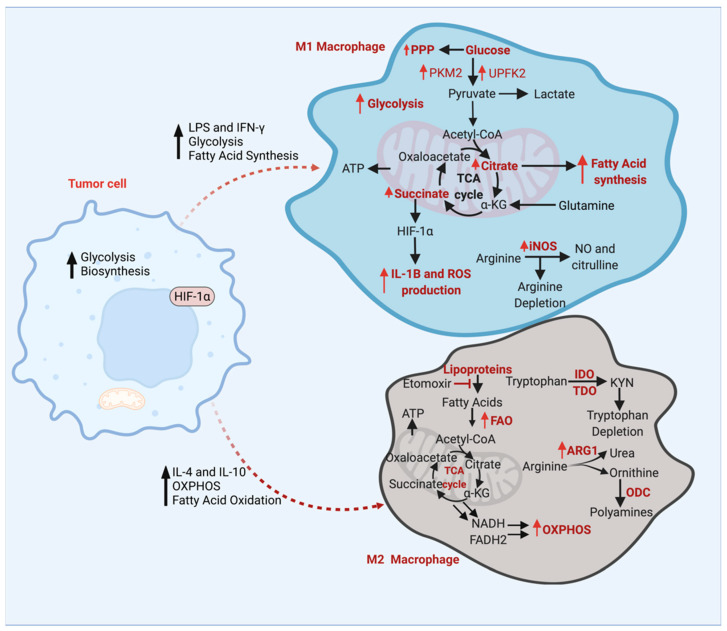
Key metabolic pathways of M1 and M2 Macrophages. M1 macrophages can first be activated by an increase in levels of LPS and IFN-γ, increase in glycolysis, and increase in fatty acid synthesis. Due to the increase in glycolysis, the enzymes PKME and UPFK2 are upregulated, making more pyruvate and increasing the glycolytic metabolism. This increase leads to an increase in PPP, creating more NADPH for ROS and NO production. The increase in pyruvate allows more acetyl CoA to enter the TCA cycle, increasing the levels of citrate and succinate. Higher citrate levels are then used in FAS. Succinate accumulation then regulates HIF1α leading to further release of IL-1ß and ROS production. Meanwhile, arginine is converted into NO and citrulline via enhanced iNOS activity, further depleting the cell’s arginine levels. M2 macrophages are activated by increased IL-4 and IL-10 levels, increased OXPHOS, and increased fatty acid oxidation. Lipoproteins go through the fatty acid oxidation process and are converted into acetyl-CoA. FAO inhibition can occur by a CPT-1 inhibitor, etomoxir. When acetyl-CoA goes through the TCA cycle, ATP, NADH, and FADH2 are made. The electron carriers NADH and FADH2 are used in OXPHOS. M2 macrophages also cause tryptophan depletion by converting tryptophan into KYN via IDO and TDO enzymes. Arginine is also converted into urea and ornithine by enhanced ARG1 activity. Ornithine then converts into polyamines using the ODC enzyme. HIF1α, hypoxia-inducible factor 1α; LPS, Lipopolysaccharide; IFN-γ, interferon-γ; OXPHOS, oxidative phosphorylation; PPP, pentose phosphate pathway; PKM2, pyruvate kinase; UPFK2, ubiquitous 6-phosphofructo-2-kinase; TCA, tricyclic cycle; ROS, reactive oxygen species; NO, nitric oxide; α-KG, alpha-ketoglutarate; iNOS, inducible nitric oxide synthase; ARG1, arginase-1; ODC, ornithine decarboxylase; IDO, indoleamine 2,3-dioxygenase; TDO, tryptophan 2,3-dioxygenase; KYN, kynurenine.

The TME is regulated by crosstalk within and across all its cellular components, including TAMs. This extensive interplay between the components often includes extracellular metabolites that act as communication signals and sources of energy. All parts of the TME also compete for the limited supply of oxygen and nutrients, shaping the metabolism of cancer cells as well as the TAMs [16]. Hanahan et al. identified two hallmarks of cancer: the reprogramming of the energy metabolism and evasion of immune destruction [62]. An altered tumor metabolism creates a TME that depletes critical metabolites, including glucose, glutamine, and tryptophan, and generates immune inhibitory metabolites, including kynurenine [63]. TAMs are also involved in the metabolic reprogramming of the tumor cells. Interleukin 1-β (IL-1β), secreted from the M2 macrophages, phosphorylates the key glycolytic enzyme glycerol-3-phosphate dehydrogenase of glioma cells which increases the substrate affinity and glycolytic metabolism of the glioma cells and promotes tumorigenesis [64]. Therefore, it is obvious that metabolism in the TME regulates the differentiation, mobilization, polarization, and anti-tumor immune responses of TAMs. Here, we discussed how different types of metabolic pathways can influence TAM survival and cellular functions (Figure 2).

### 3.1. Glucose Metabolism of TAMs 

Lipopolysaccharide (LPS)/IFN-gamma-stimulated M1 macrophages require elevated glycolysis and fatty acid synthesis; meanwhile, IL4/IL-10-activated M2 macrophages require enhanced fatty acid oxidation (FAO) and oxidative phosphorylation (OXPHOS) [64,65]. In cardio-metabolic diseases, the inhibition of FAO or lipolysis by etomoxir (a carnitine palmitoyl transferase 1 inhibitor) resulted in impaired M2 activation [66,67]. M1 macrophage metabolism is classified by increased glycolysis, an enhanced flow of glucose through the pentose phosphate pathway (PPP), fatty acid synthesis (FAS), and the truncated citric acid cycle (TCA cycle) with increased levels of succinate and citrate [68,69,70]. The PKM2 enzyme is another factor that contributes to TAMs polarization [71]. Pyruvate kinase is a glycolytic pathway enzyme that catalyzes the conversion of phosphoenolpyruvate (PEP) to pyruvate [72]. PKM2 is an isoform of pyruvate kinase and is highly expressed in proliferating cells and exists in either an enzymatic monomer or dimer [73]. The PKM2 dimer moves into a cell’s nucleus and interacts with hypoxia-inducible factor 1α (HIF1α). This PKM2–HIF1α complex then binds to the promoter of IL-1ß and inhibits this proinflammatory cytokine, resulting in an increased glycolytic flux and promoting M2 polarization [71,74]. PKM2 dimerization increases the glycolytic flux, which then allows TAMs to utilize oxidative phosphorylation, and promotes the transformation of TAMs from M1 to M2, thereby increasing the number of M2 macrophages and hindering the anti-tumoral immune response [71,75]. Therefore, the inhibition of the pyruvate kinase isoform PKM2 attenuates M2 polarization while activating the M1 polarization of the TAMs. 

IFN-γ and LPS stimulation upregulate phosphofructokinase 2 (PFK2) into its ubiquitous isoform, ubiquitous 6-phosphofructo-2-kinase (U-PFK2). U-PFK2 converts fructose-6-phosphate (F6P) into fructose-2,6-bisphosphate (F2,6BP), activating enzyme phosphofructosekinase-1 (PFK1) [65]. U-PFK2 expression is modulated by HIF1α, leading to the high glycolytic metabolism that M1 macrophages acquire [65,70,72,76]. Downregulation of hexokinase, a glycolytic enzyme, can inhibit the formation of inflammasomes (which secrete proinflammatory cytokines) in macrophages after LPS activation [76]. The inhibition or deletion of glycolytic enzyme pyruvate kinase can shunt M1 phenotype activation even with LPS and IFN-γ stimulation [71]. The upregulation of glycolysis and changes in the TCA cycle metabolic intermediates can be beneficial for M1 macrophage activation. The accumulation of the TCA cycle intermediates, succinates, activates hypoxia-inducible factor 1α (HIF1α), leading to the upregulation of glycolysis, interleukin-1ß, and reactive oxygen species (ROS) production [77]. Succinate accumulation in tumor cells and macrophages was reported to facilitate tumorigenesis, cancer cell migration and metastasis [78]. This increase in succinate is attributed to a reduced succinate dehydrogenase (SDH) activity. Succinate accumulation triggers PI3K-HIF-1α-mediated pro-tumor TAM polarization and cancer metastasis and serves as a biomarker in lung cancer [78]. Thus, TAMs display a high degree of metabolic plasticity in the glucose metabolism and response to metabolic cues present in the TME. 

### 3.2. Amino Acid Metabolism

In the TME, specific amino acid metabolites, including tryptophan and arginine, activate TAMs, and these activated TAMs metabolize higher amounts of tryptophan and arginine [69,79,80,81]. The arginine is highly metabolized in TAMs, resulting in arginine depletion within the TME. Arginase-1 (ARG1), the first enzyme of the arginine metabolism, is highly expressed in M2 TAMs [82,83]. Arginine is metabolized by arginase enzymes to ornithine and urea, and by nitric oxide synthase (iNOS) to nitric oxide (NO) and citrulline [77]. Pro-inflammatory cytokines inhibit ARG1 activity, yet activate iNOS activity [81]. In the urea cycle, ARG1 converts arginine into ornithine and urea, which are then converted to polyamines by orthenine decarboxylase (ODC). ODC is highly expressed in tumors and promotes tumor development. In prostate cancer, ARG1 along with ODC can reduce iNOS activity and NO concentration by depleting arginine as a substrate. Pro-tumoral M2 macrophages show more arginase activity with more polyamine synthesis, leading to a higher cell proliferation, while antitumoral M1 macrophages have more iNOS activity, leading to decreased cell growth [81]. The deletion of NO and arginine is related to tumorigenesis and produces pro-tumoral growth factors in the TME, while also reducing the arginine concentration to suppress immune function [81]. Tryptophan is an essential amino acid, metabolized into kynurenine (KYN) to a high degree in TAMs by the tryptophan metabolizing enzymes Indoleamine 2,3-dioxygenase (IDO) and tryptophan 2,3-dioxygenase (TDO). KYN plays a role in repressing the antitumor immune responses regulated by the aryl hydrocarbon receptor (AHR), a member of a transcription factor family that activates xenobiotics including KYN [84]. Elevated AHR levels by TDO and IDO cause an increase in AHR target gene transcription, which serves as the antitumor targets. TAMs also express arginase and iNOS enzymes, which lead to arginine depletion, as well as IDO elevation, which causes tryptophan depletion [63]. A reduced level of arginine and tryptophan promotes metabolic quiescence, resulting in the temporary stoppage of cell cycle progression [85].

While arginine and tryptophan depletion can activate TAMs, the inhibition of the glutamine metabolism of the immunosuppressive myeloid cells converted these cells into inflammatory macrophages [63]. Glutamine synthetase (GS) is a glutamate-metabolizing enzyme that converts glutamine from ammonia and glutamate, two substrates that show toxicity to the central nervous system (CNS) and can cause neuroinflammation [70,86]. GS enzyme activity is highly correlated with TAM polarization to immunosuppressive M2 macrophages in TME [87]. Palmieri et al. show that the genetic deletion of the GS enzyme in TAMs leads to elevated glutamate levels in TAMs [70]. GS activation promotes the transition of monocytes to M2 macrophages, while GS inhibition causes the phenotypes to switch to M1 macrophages, causing increased succinate levels [63,70,86,88]. Elevated succinates in macrophages regulate pro-inflammatory responses by inhibiting anti-inflammatory cytokines and promoting HIF-1α stabilization [70]. M2 macrophages express more GS, resulting in higher levels of glutamine synthesis than M1 macrophages. Thus, GS and glutamine depletion in TAMs show M1 polarization with reduced tumor metastasis, while the depletion of other amino acid metabolites, including arginine and tryptophan, can activate immunosuppressive TAMs. 

### 3.3. Fatty Acid Metabolism 

TAMs, which resemble M2 macrophages, increase fatty acid uptake and fatty acid oxidation [67,89,90]. The inhibition of lipid uptake through the blocking of CD36, a glycoprotein that functions in oxidized LDL uptake and foam cell formation, and FAO inhibition can shut off the switch to the TAM phenotype in the TME [91]. CD36 has been proposed to be a marker for different types of epithelial cancers, and it has been confirmed that CD36 is highly expressed in the TME, especially in macrophages, and highly correlates with the FAO gene signature [91]. A deficiency in monoacylglycerol lipase (MGLL) can cause lipid accumulation in TAMs of colorectal cancer, resulting in M2 macrophage activation and inhibiting CD8^+^ T cell function [92]. Ding et al. examined how the deficiency in angiopoietin-like 4 (ANGPTL4), which inhibits lipoprotein lipase (LPL), can lead to enhanced fatty acid synthesis (FAS) and diminished fatty acid oxidation (FAO) in adipose tissues and macrophages [93]. The dysregulation of fatty acid metabolism induces ER stress and inflammation, leading to an upregulation of glycolysis and fatty acid synthesis which inhibits lipoprotein lipase (LPL) [91]. TAMs thus sustain their fatty acid supply by the phagocytosis of extracellular fatty acids, increased LPL activity, and de novo production of fatty acids from acetyl CoA by fatty acid synthase [90]. All of these resources can be utilized to activate tumor-promoting M2 TAMs. Lipid droplets accumulated in the macrophages regulate the catabolism of unsaturated fatty acids for mitochondrial respiration and polarize TAMs into M2 phenotypes [90]. The inhibition of LD accumulation and the disruption of LD synthesis in myeloid cells showed a decreased tumor growth in colon cancer patients [90], pointing to the role of LD accumulation in TAMs favoring the tumor growth. Although previous studies have mentioned that long chain fatty acid (LCFA) oxidation can lead to the activation of M2 macrophages; a recent study showed that LCFA oxidation and oxidative phosphorylation are not required for M2 macrophage polarization [94]. However, complex I of electron transport chain inhibitors can inhibit the expression of pro-IL-1ß in LPS-stimulated macrophages, suggesting that a redox signal, potentially increased ROS, from complex-I can trigger M2 polarization [72,95]. Thus, TAM activation can be due to limited FAO and increased FA synthesis, eventually leading to high levels of lipid droplets and acetyl CoA production.

### 3.4. Tumor Acidity in Dysregulated TAM Metabolism 

A key feature of the TME is extracellular acidosis and intracellular alkalization due to the local drop of pH, caused by increased lactate-mediated acidification [96]. In low pH environments, anti-tumor effectors, including T cells and NK cells, lose their function, while immunosuppressive effectors, such as myeloid cells and regulatory T cells, can sustain tumor progression and block antitumor immune responses [97]. Cancer cells are highly glycolytic, which increases lactate dehydrogenase (LDH) activity, converts pyruvate to lactate, and enhances lactate generation [96,98]. Tumor cells are essentially net producers of acid due to aerobic glycolysis, producing large amounts of carbon dioxide and lactic acid [97]. Lactate accumulation can be a significant TME signal to polarize M2 macrophages [99]. As tumors grow, glucose is depleted, and lactate concentrations increase. This drives M2 macrophage reprogramming, and they need to use lactate as a source of oxidative phosphorylation, which then results in high TCA activity [99]. Even in the presence of high glucose and lactate levels, TAMs use lactate for citrate production because pyruvate generation from lactate requires fewer enzymatic processes and produces NADH [99].

Cancer cells exploit different proton pumps that cotransport lactate and protons. These pH sensing proteins signal cancer cells to promote their survival in the low pH environment. G-protein coupled receptors (GPCRs) are examples of pH sensing proteins and when the TME is acidic, they increase cAMP production and cancer cell survival [97]. With that, myeloid cells are also very sensitive and can detect even the smallest changes in local pH and then instantly respond by polarizing and changing their activity [97]. TME acidity is a major attractor of myeloid cells because immune response molecules, including selectins, can be modified by acidity. Selectin regulates the recruitment of leukocytes to inflammatory sites, and under low pH conditions, P and L-selectins bind in high affinity to a P-selectin glycoprotein ligand 1 (PSGL-1), leading to increased monocyte adhesion to the endothelium [97]. An acidic environment can also induce proinflammatory mediators in different tumor-associated myeloid cells. The enzyme iNOS is heavily activated in macrophages through a process that includes the increased nuclear factor k-light-chain-enhancer of B cells’ (NF-kB) translocation. NO can increase the acid-loading by impairing the intracellular pH recovery [97,100]. This extracellular acidity can increase triglyceride storage in macrophages, promoting M2 TAM activation [100]. Thus, a more acidic extracellular TME can lead to macrophage polarization into M2 activation. This dysregulation of pH seen in tumor cells can drive different processes including tumorigenesis, and the progression, migration, and reprogramming of cancer cell metabolism. 

### 3.5. The Effect of TAMs Metabolism on T Lymphocytes 

The amino acid metabolism of TAMs affects T cells’ immune response [16]. Arginine is necessary for T cell development and activation since the intracellular arginine concentration of the T cells affects the metabolism, function, and anti-tumoral response of T cells [69,79,80]. However, as the arginine metabolic pathway plays a vital role in M2 TAMs’ functions, increased ARG-1 activity by M2 macrophages creates an arginine-depleted microenvironment which negatively affects T cells’ activation and proliferation, decreasing T cells’ anti-tumor function [87]. Moreover, tryptophan and its metabolic product kynurenine affect the immune response against tumor cells [101]. Elevated IDO enzyme activity in the TAMs converts tryptophan into kynurenine [87]. The functions of T cells are disrupted not only by the presence of kynurenine, but also due to the diminution of tryptophan within the TME [102]. Additionally, kynurenine induces the death of T cells, lessening the pro-inflammatory T lymphocyte count, and thereby diminishes the immune response of T cells [103]. As a result, the increasing tryptophan metabolism of the TAMs dampens the T cell immune response in various ways, providing an opportunity for the progression and spread of tumors [87]. However, the tryptophan metabolism and the association between T cells and TAMs are more complex. Zhao et al. showed that the IDO enzyme activity in TAMs of the hepatocellular carcinoma tissue is being boosted by CD69^+^ T cells, in which CD69 is expressed on early-activated T cells within the TME [104]. TAMs affect the circulating CD69^-^ T cells by promoting CD69 expression, which draws them into the TME. The early-activated CD69^+^ T cells secrete inflammatory cytokines such as IFN-γ [105] which causes the elevation of the IDO enzyme in TAMs and expedites tryptophan metabolism [106]. Thus, increased IDO activity in TAMs leads to the suppression of an anti-tumoral T cell response by blocking the inflammatory cytokines’ release and proliferation of anti-tumoral T cells [105]. Taken together, when these amino acids, specifically arginine and tryptophan, are depleted, T cell activation and proliferation are inhibited, while TAMs are activated [107]. 

The activation of TAMs’ M1 dominancy in tumor tissue leads to enhanced inflammatory T cell infiltration and promotes anti-tumoral T cell response [70]. Glutaminolysis or the breakdown of glutamine can cause an inflammatory anti-tumor response in effector T cells, and an increased glutamine consumption polarizes macrophage M1 activation [70]. Palmieri et al. show that the genetic deletion of the GS enzyme in TAMs leads to elevated glutamate levels in TAMs [70]. Glutamate can be converted to a TCA cycle intermediate succinate [68,70]. An increased succinate accumulation in TAMs triggers the TAMs phenotype shift from M2 to M1. Therefore, the high activity of the GS enzyme causes an increased glutamate metabolism [70]. TAM polarization to M2 phenotype activation negatively impacts T lymphocytes’ anti-tumoral activity [16] and results in tumor cells escaping from T cell-mediated immune activity [70]. However, M2 cells rely on OXPHOS for energy metabolism [108], and use amino acids and fatty acids as substrates for the TCA cycle [109] and express low glucose transporter (GLUT-1) [110]. GLUT-1 deleted macrophages use amino acids and fatty acids as an energy source alternative to glucose, leading to the depletion of amino acids in the TME and leaving T cells with insufficient amino acids to maintain their functions [111]. Taken together, amino acid metabolic pathways and the GLUT-1 transporter influence TAM polarization, survival, and cellular functions. There are strong connections between TAM metabolism and the overall immune response against tumors which is more often beyond the M1/M2 classification. Therefore, TAMs’ metabolic pathways are valuable targets for cancer therapy. 

## 4. TAM Metabolism as a Target for Immunotherapy 

Rapid tumor growth of the tumor tissue causes an increased metabolic demand of tumor cells, which creates angiogenesis in the TME [112]. However, newly formed vessels in the tumor tissue only supply insufficient oxygen because of their disrupted structures, which creates low perfusion and a hypoxic TME [113]. Consequently, by changing their metabolic preferences, the cells in the TME try to adapt to this hostile condition characterized by hypoxia and nutrient deprivation [114]. TAMs mainly polarize to immune suppressive M2 macrophages in the TME and often use oxidative phosphorylation for energy metabolism [115]. By shifting their energy metabolism to OXPHOS, TAMs can use other substrates for energy sources, such as amino acids and fatty acids, by adding those substrates’ products to the TCA cycle [11,16]. Here, we discuss the importance of targeting TAMs’ metabolic pathways to improve the immunotherapies against cancers.

### 4.1. Targeting TAM Metabolism for Improving Cancer Therapy

Due to the strong relation between the TAMs’ metabolism and anti-tumoral immune response, targeting the TAMs’ metabolism is a considerable approach for cancer therapy. The bioenergetic metabolic pathways of TAM subtypes for energy generation differ from each other [16], with the metabolism shift to oxidative phosphorylation facilitating the pro-tumoral M2 polarization of TAMs [11]. Therefore, blocking the TAM metabolic shift to oxidative phosphorylation has the potential to reverse the immune suppressive effects of the M2 TAMs within the TME [116]. Chen et al. showed that the elevated activity of the Mammalian Target of rapamycin (mTOR) pathway enhances the M2 polarization of TAMs in a mouse model of colon cancer [117,118]. Metformin, an mTOR-inhibiting drug, hinders the M2 polarization of macrophages [119]. Moreover, lactate plays an important role in TAMs polarization, and its high concentrations in the TME promote the M2 polarization of the TAMs. Owing to the direct link between lactate concentration and TAM polarization, lactate can be targeted to manipulate the TAMs’ metabolism. Lactate dehydrogenase inhibitor decreased lactate production in the TME to increase the M1/M2 ratio, thereby reestablishing the anti-tumoral activity of T lymphocytes [120]. The restoration of the lactate dehydrogenase B activity showed reduced breast cancer cell proliferation by limiting the macrophage-derived lactate utilized by the cancer cells [121,122]. Since the GS enzyme triggers TAMs’ polarization by increasing the succinate concentrations in TAMs, it is a contemporary target considering its effect on amino acid metabolism [87]. The GS-blocking drug (methionine sulfoximine (MSO) has been used to transform pro-tumoral M2 macrophages into anti-tumoral M1 macrophages, which enhances anti-tumoral T-helper cells and cytotoxic T cells and reduces the activity of immune-suppressive T-regulatory cells, ultimately repressing the tumor growth in mice with Lewis lung carcinoma [70]. A recent report by Selvanesan et al. showed that gemcitabine (GEM) in combination with immunotherapy altered the immunosuppressive TME in a mouse model of PDAC. GEM, which showed to increase ROS production, when combined with the tetanus toxin, decreased the immunosuppression of the PDAC tumor by decreasing the MDSC, and Trges while increasing the CD4^+^ T cells [123].

Fatty acids are oxidized in the mitochondria, and this oxidization process provides substrates for the oxidative TCA cycle [124]. In addition, fatty acid oxidation is shown to trigger the M2 polarization of TAMs [91]. Considering the relation between FAO oxidation and the M2 activation of TAMS, it became a new target for immunotherapy in cancer patients. Blocking FA oxidation in the mitochondria with FAO-inhibiting drugs (etomoxir, ranolazine) decreases the substrate supply to the TCA cycle, which significantly can mitigate the oxidative phosphorylation of M2 macrophages and blunt immune-suppressive M2 macrophage activity. Additionally, the blockage of M2 activity with FAO-inhibiting drug results in decreased immune-suppressive cytokines production. Hossain et al. showed that FAO inhibition with etomoxir does not cause increased anti-tumoral T cell function. However, the combination of etomoxir with chemotherapy re-establishes the T cells’ anti-tumoral functions [89].

As amino acids have a vital role in the polarization of TAMs, amino acid metabolism is another significant target for cancer therapy. The IDO enzyme, which metabolizes tryptophan, is one of the targets [125] since the IDO enzyme and tryptophan metabolites (especially kynurenine) favor the tumor growth by blocking the anti-tumoral immune response [126]. Several studies were reported to develop IDO-blocking drugs [127,128,129] and some of the drugs reduced the IDO activity in tumor-associated macrophages [128,129]. Yue et al. showed that tumor growth is decreased in CT26 tumor-bearing mice [128]. One of the main therapeutic targets for cancer treatment is the inhibition of arginine metabolism by blocking the arginase enzyme in TAMs [130,131]. The arginase enzyme inhibitor CB-1158 molecule is an oral agent that blocks arginase enzyme activity and has been tested in a clinical trial (NCT02903914) [132]. Blocking arginase activity in TAMs with CB-1158 causes increased arginine levels in the TME. With a high arginine supply, T cells metabolize arginine and maintain their anti-tumoral functions [107]. Taken together, targeting the metabolism of TAMs emerges as a new strategy to reinvigorate the T cells’ anti-tumor immune function (Table 1). 

**Table 1:** Selected agents targeting TAM metabolism for the treatment of cancers.

Drug	Target	Metabolic Effect	Disease	Effect on TAMs and TME
Metformin	AMPK (AMP-activated protein kinase)	mTOR inhibition, increased lipid breakdown, impaired glycolysis	Cancer	Decreased M2 polarization of macrophages [133,134]
Compound 1	Lactate DHG	Decreased lactate production, interfering TME acidity	Cancer	Increased M1/M2 ratio in TME [120]
Methionine Sulfoximine	Glutamine Synthetase	Inhibition of glutamine metabolism, impaired glutamine usage by TAMs	Lewis Lung Carcinoma	Repolarization of M2 macrophages to M1 macrophages [70]
Etomoxir	FAO	Impaired lipid oxidation	Cancer	Decreased M2 activity [89]
Ranolozin	FAO	Impaired lipid oxidation	Cancer	Decreased M2 activity [89]
CB-1158, L-Norvaline	Arginase	Inhibition of arginine synthesis, decreasing arginine levels	Solid tumors, Alzheimer’s disease	Increased anti-tumoral T cell response in TME, Repolarization of TAMs [89]
Doxorubicin, Paclitaxel, Methodextrate	ABC Transporter	Disruption of efflux mechanism of cells leading to lipid accumulation in cells	Multidrug Resistant Cancer	Repolarization of TAMs [135]
Pralnacasan, NCX-4016, YVAD, VAD	Caspase-1	Decreased lipid levels in inflammatory cells	Cancer, autoimmune diseases, rheumatoid arthritis, osteoarthritis	TAMs repolarization [136]
Rapamycin, RAD001	mTOR	Decreased synthesis of proteins and nucleic acids	Amyotrophic lateral sclerosis, Glioma, Non-small cell lung cancer	Repolarization of TAMs [137]
PHGDH inhibitors	Phosphoglycerate dehydrogenase	Disruption of serine synthesis	Breast cancer, lung cancer, malign melanoma	Increased anti-tumoral T cell response in TME [138,139,140]
2-deoxyglucose (2-DG)	Hexokinase 2	Aerobic glycolysis inhibition by effecting the first step of glycolysis	Cancer	Repolarization of TAMs [141]
CB-839	Glutaminase-1	Negative effect on glutamine metabolism	Breast Cancer	Decreased glutamate levels in TME [142,143]

### 4.2. Na^+^/H^+^ Exchanger (NHE1) as a Modulator of TAM Metabolism and Immune Checkpoint Therapy 

**Figure 3:**
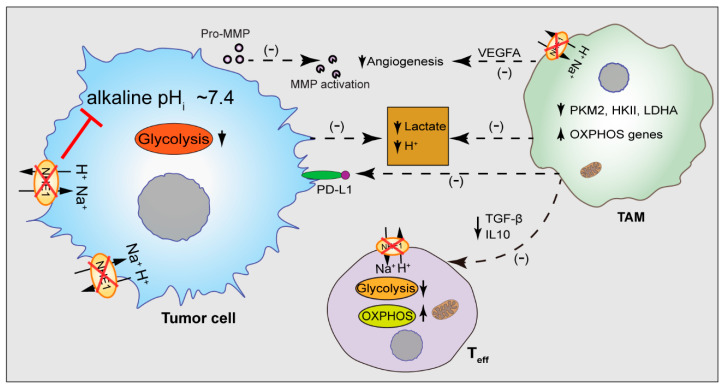
NHE1 blockade rewires TAMs metabolism. Blockade of Na/H exchanger (NHE1) affects tumor progression via multiple mechanisms. NHE1 blockade reduces glycolysis in the tumor cells and the macrophages reducing H^+^ and lactate in the TME. Alteration of acidic pH_e_ reduces tumor angiogenesis by blocking metalloprotease (MMP) activation. Reduced NHE1 activity in the TME reprograms macrophage metabolism by increasing OXPHOS genes while reducing glycolytic genes of the macrophages, resulting in reduced TGF-β and IL10 secretion which increases T cell effector function and reduces regulatory T cells. Reduced PKM2 expression of macrophages leads to reduced PD-L1 expression of the cancer cells. Therefore, blocking NHE1 rewires the metabolism of the TAMs which improves the anti-tumor immunity of the T cells. MMP, matrix metalloproteinase; VEGF, vascular endothelial growth factor; PKM2, pyruvate kinase M2; HKII, hexokinase II; LDHA, lactate dehydrogenase; PD-L1, programmed death-ligand 1.

The Na/H exchanger regulates the cellular pH hemostasis in all cell types [144] which expels H^+^ ions out of cells in exchange for a Na^+^ ion in maintaining the alkaline intracellular pH necessary for cell survival [145]. The high glycolytic metabolism of the tumor cells leads to cytosolic H^+^ accumulation and the direct consequence of glycolytic metabolism is the activation of the NHE1 function. The expression of a glycolytic enzyme hexokinase 2 (HKII) correlates with high NHE1 activity in rat fibroblasts and murine T helper cells [146,147]. Additionally, the NHE1 protein has been shown to play an important role in the progression of breast cancer, glioma, and ovarian cancer [95,144,148,149,150]. However, NHE1 activity is also prominent in the TME where it was shown to affect TAM polarization and metabolism. The pharmacological inhibition or transgenic deletion of NHE1 in TAMs alone or in combination with the alkylating agent temozolomide shifts the TAMs metabolism to OXPHOS, increases inflammatory macrophages, and favors the anti-tumor function of the TAMs and T cells [95,148,151]. Additionally, the blockade of NHE1 with HOE642 increases the glucose uptake of the glioma-infiltrating CD11b^+^ macrophages with metabolic shifts towards the OXPHOS metabolism, and favors the anti-tumor macrophage function [95]. TAMs promote the expression of PD-L1 in the pancreatic ductal adenocarcinoma cells and are associated with the high expression of the key glycolytic enzyme, pyruvate kinase M2 (PKM2), as assessed by analyzing the PDAC cohort from the TCGA database [152]. Interestingly, in bladder cancer cells, a reduced NHE1 gene expression is associated with a reduced PKM2 gene expression [153], pointing to the role of the NHE1 protein in the glycolytic metabolism, and PD-L1 expression. Therefore, targeting the NHE1 protein may reprogram the metabolism and phenotype of immune cells in the TME, potentially improving the therapeutic efficacy of the immune checkpoint therapy (Figure 3). 

### 4.3. Targeting TAM Metabolism to Improve Immune Checkpoint Therapy 

**Figure 4:**
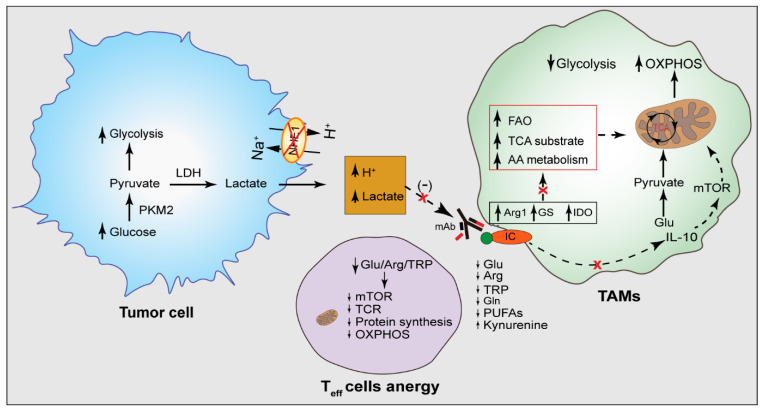
TAMs metabolism as a target for immunotherapy. Highly proliferative cancer cells use aerobic glycolysis and consume an elevated amount of glucose for bioenergetic and biosynthetic purposes releasing lactate and H^+^ in the TME. In addition to affecting cellular signaling, acidic TME also affects therapeutic antibody function and integrity and may cause breakage of the antibodies. Immune checkpoint molecules cause elevation of IL-10 signaling which increases the TCA substrates and OXPHOS metabolism, and promotes TAM’s tumor promoting function. The elevated level of Agr1, GS, and IDO cause elevated flux of TCA cycle substrates via amino acid metabolism and FAO, resulting in increased OXPHOS metabolism, a hallmark of tumor-promoting TAMs. These lead to depletion of metabolites in the TME necessary for T cell’s effector function. Reducing TME acidity may increase the OXPHOS genes which elevates the OXPHOS metabolism, reduces glycolysis, and promotes TAM’s anti-tumor function. As the M1/M2 classification is overly simplified, the preference of glycolysis for M1 TAMs and OXPHOS for M2 TAMs are also simplified. Therefore, the increase of TAM’s OXPHOS, because of glucose metabolism to pyruvate and its progression to the TCA cycle, may increase the TAM’s anti-tumor function. Additionally, targeting Arg1, GS, and IDO, and reducing tumor acidity by blocking NHE1 activity may increase the key metabolites in the TME necessary for the T cells’ effector function. Glu, glucose; ARG1, arginase 1; IDO, indoleamine 2,3-dioxygenase 1; GS, glutamine synthase; AA, amino acids; FAO, fatty acid oxidation; PKM2, pyruvate kinase M2; LDH, lactate dehydrogenase; TCR, T cell receptor; mTOR, mammalian target of rapamycin; IC, immune checkpoint; mAb, monoclonal antibody.

The metabolism of TAMs is highly heterogeneous not only within different TAM populations, but also among similar TAM populations which, at least in part, explains the failure or sub-optimal efficacies of TAM-directed therapeutic approaches and immune checkpoint blockade therapies [154,155]. Pro-tumoral M2 macrophages secrete anti-inflammatory cytokines such as IL-10 and TGF-Beta to block the anti-tumoral T cell response against tumoral cells [156]. Inflammatory M1-activated macrophages utilize the glycolytic metabolism for their function [124] and the PKM2 in macrophages enables the metabolic shifts of the macrophages from OXPHOS to glycolysis. PKM2 has been shown to enhance the expression of PD-L1 in the LPS-activated macrophages and colon cancer-infiltrating CD1b^+^CD4/80^+^ macrophages [71]; targeting the PKM2 with a synthetic compound, Tepp-46, reduced the PD-L1 expression in macrophages and dendritic cells [71]. Chen et al. showed that transcriptomic analysis of the myeloid cells upon leukocyte immunoglobulin-like receptor B (LILRB2) antagonism shows changes in the metabolic genes and gene networks associated with inflammatory myeloid cells, as opposed to their alternatively activated phenotype [157]. The LILRB2 blockade by an antibody transformed the tumor-infiltrating myeloid cells in the non-small-cell lung carcinoma tumor tissues into the inflammatory activation spectrum and increased the efficacy of the immune checkpoint blockade therapy [157]. Moreover, the deletion of an immune checkpoint brake protein TNFAIP8L2 (TIPE2) in macrophages [158] leads to increased mitochondrial respiration and inflammatory IFN-γ signaling [159]. In melanoma, Strauss et al. showed the connection between myeloid cell’s PD-1 expression and metabolic reprogramming of the myeloid progenitor cells [160], where PD-1-deficient myeloid cells induced increased metabolic intermediates of glycolysis, the pentose phosphate pathway, and the TCA cycle, but, most prominently, elevated the level of cholesterol, resulting in an increased T cell effector function [160]. Tim3, an immune checkpoint molecule, controls the glycolytic metabolism and lactate production by macrophage cells [161,162]. However, some TAM subsets rely on PD-L1/PD-1 signaling to stimulate glycolysis. Therefore, the efficacy of targeting immune checkpoint molecules relies not only on the T cells’ functional reinvigoration, but also on the metabolic preference of the TAMs. The inhibition of tumor cells’ glycolysis ensures the sufficient glucose necessary for TAM function. Ultimately, targeting the TAMs’ PD-L1/PD-1 signaling may restore the M1-like phenotype, at least partially [16]. Taken together, targeting the TAMs and tumor metabolism in combination with immune checkpoint blockade therapy in a multifaceted manner may increase their anti-cancer efficacy (Figure 4). 

### 4.4. Effect of Tumor Acidity on the Activity of Therapeutic Antibodies 

Due to the elevated aerobic glycolysis in tumor cells, lactate and H^+^ accumulate in the TME making an acidic TME that directly blunts T cells’ anti-tumoral response and therapeutic antibodies’ activity [97,163,164]. A low pH induces the TAMs’ transformation to immune-suppressive M2 macrophages, which blocks the anti-tumoral T cell response [97]. As T cell activity is required for immune-checkpoint antibodies’ efficacy, the depleted anti-tumoral T cell response is one of the underlying factors that interfere with immunotherapy efficacy [97,163]. It has been shown that the administration of alkalinizing agents to colorectal cancer patients increased the pH of the TME, resulting in the reversal of the lessened anti-tumoral T cell response stemming from a low pH [165]. The administration of alkalinizing agents significantly increased the overall response to the therapeutic antibodies along with the resurgence of an anti-tumoral T cell response [165]. The acidic pH of the TME negatively affected the therapeutic antibodies by the breakdown of antibodies and damaging their binding capacities to the cell-surface targets [97]. Additionally, Pilon-Thomas et al. showed that when mice bearing melanoma were treated with anti-CTLA-4 and anti-PD-1 antibodies, in combination with bicarbonate to increase the pH in the TME, the therapeutic efficacy of the immune checkpoint inhibitors increased significantly [166]. It is well known that an acidic pH affects therapeutic antibodies’ binding to the targeted complex negatively [167]. However, an acidic TME can be exploited by selectively targeting the TME with engineered or conditionally active therapeutic antibodies possessing enhanced binding and efficacy in the acidic TME [168]. Engineered trastuzumab, which is a HER2^+^ selective monoclonal antibody, showed better binding and efficacy at pH 6.5 than those at pH 7.5, indicating the increased efficacy in the acidic environment, whereas the toxicity in the healthy tissue was diminished. Therefore, although an acidic TME affects the efficacy of the immune checkpoint targeted antibodies, it also presents a unique advantage in targeting tumors featuring an acidic TME while reducing systemic toxicity. 

## 5. Conclusions

Once cancer was recognized as a metabolic disease and it was known that cancer cells utilize aerobic glycolysis to meet the energy demand. Similar to cancer cells, TAMs also reprogram their metabolism depending on the metabolic cues they encounter and shape the neoplasm. Hence, the macrophage is recognized as a dynamic cell population with various activation phenotypes and metabolic signatures in the different phases of cancer development. The macrophages’ adaptation to metabolism has been investigated in recent years, and their functions are intimately connected to the metabolic state. Current macrophage targeting therapies, aiming at the alteration or depletion of the M2 macrophages, often fail due to innate and acquired resistance to the therapy and developing alternative immunosuppressive cells compensating for TAMs. The most durable approach would be targeting multiple immunosuppressive pathways in combination with the metabolic reprogramming of the macrophages. However, the challenge is to reduce the immunosuppressive function of the immune cells and, at the same time, provide immunostimulatory cues. New studies are required to evaluate the immunomodulatory impact of anti-cancer therapies, especially the new generation of immunotherapies and their interplay with the metabolism of different TME components.
